# The Role of miRNAs as Early Biomarkers in Obesity-Related Glomerulopathy: Implications for Early Detection and Treatment

**DOI:** 10.3390/biomedicines13051030

**Published:** 2025-04-24

**Authors:** Maruja Navarro-Díaz, Marina López-Martínez

**Affiliations:** 1 Genomic Platform, Germans Trias i Pujol’s Research Insitute, 08916 Badalona, Spain; 2 Nephrology Department, Sant Joan Despí Moisès Broggi Hospital, 08970 Sant Joan Despí, Spain; 3 Nephrology Department, Vall d’Hebron University Hospital, 08035 Barcelona, Spain; marina.lmga@gmail.com; 4 Department of Medicine, Universitat Autònoma de Barcelona, 08193 Bellaterra, Spain

**Keywords:** microRNA, biomarker, obesity-related glomerulopathy

## Abstract

Obesity increases the risk of cardiovascular disease, diabetes and chronic kidney disease. Obesity-related glomerulopathy (ORG) is a potentially reversible cause of kidney disease that often progresses silently until it reaches irreversible stages. However, we are still lacking a sensitive and specific biomarker to identify patients with obesity who are at risk of developing CKD. The role of microRNAs (miRNAs) has emerged as a promising area for diagnostic and therapeutic applications in kidney disease. Recent research has highlighted the specific roles of various miRNAs in renal function, showing that their dysregulation can contribute to the development of kidney diseases. This review will discuss the emerging role of miRNAs in the context of ORG, focusing on their potential as biomarkers and therapeutic targets for this increasingly prevalent disease.

## 1. Introduction

Obesity-related glomerulopathy (ORG) is a potentially reversible cause of kidney disease that often progresses silently until it reaches irreversible stages [1,2,3,4]. Beyond renal damage, obesity increases the risk of cardiovascular disease, diabetes, dyslipidemia and hypertension [5]. Consequently, there is an increased risk of overall cardiovascular morbidity and mortality [5,6].

Although its histological features were initially described by Weisinger111 and Cohen222 in the 1970s, ORG was only recognized as a distinct clinical condition in the early 2000s in patients with obesity and proteinuria, with or without renal insufficiency [3].

Subsequent findings—such as histological damage in severely obese patients without overt kidney symptoms—have helped to define the early stages of the disease. The global rise in obesity has been mirrored by a growing prevalence of ORG [4], underlining the need for earlier detection and a better understanding of its pathophysiology.

ORG is a distinct form of focal segmental glomerulosclerosis (FSGS) that arises as a pathological consequence of obesity. This glomerular lesion is the primary driver of progressive renal dysfunction in obese patients with ORG, ultimately leading to end-stage renal disease. Our research group identified histopathological renal alterations in patients with severe obesity who exhibited preserved renal function [7], demonstrating that structural renal injury occurs even in the absence of overt renal impairment or albuminuria. These findings allowed us to characterize the early stages of ORG, which predominantly manifest as glomerular alterations preceding the development of FSGS. Currently, the most commonly used biomarker for detecting renal involvement in obesity is albuminuria. However, as albuminuria lacks the sensitivity needed to detect early damage, it should be considered a late biomarker rather than an early indicator of disease onset. The study of microRNAs (miNRAs) holds promise for identifying obese patients with subclinical renal injury, specifically at a stage preceding FSGS and albuminuria. Early detection would enable timely therapeutic intervention, potentially halting disease progression and preventing the onset of chronic kidney disease (CKD), thus allowing for treatment during a reversible phase of ORG. To further investigate early-stage ORG, we developed an experimental model of obesity using Wistar rats, which exhibited incipient glomerular pathology. Our study demonstrated that rats with early-stage ORG exhibited downregulation of miRNA-205, a microRNA implicated in the regulation of PTEN, a key gene involved in podocyte lipid endocytosis [8].

Extensive research has been dedicated to clarifying the pathophysiological mechanisms underlying ORG. However, significant questions remain unanswered, such as why some individuals with obesity develop renal disease while others do not [9,10]. Although hyperfiltration has been said to be one of the first manifestations of ORG, caused by an increase in intraglomerular pressure, not all subjects with obesity that hyperfiltrate will develop overt ORG and CKD. Regardless, hyperfiltration usually passes unnoticed in clinical practice when the estimated glomerular filtration rate (eGFR) is used [11].

Clinical trials and human studies can be associated with ethical concerns, involving pharmaceutical interests and high economic expenditures. Additionally, elucidating the pathways involved in early stages of diseases and the histological effects of various conditions often requires invasive techniques that cannot be performed in humans. For these reasons, experimental research is the first step in deepening our understanding of disease development. Rodents are widely used as experimental models for preclinical studies in nephrology research. Murine models have become the system of choice, due to their low cost, fecundity, short gestation times and tractability for genetic manipulation [12,13].

The advent of new technologies in the 21st century, such as bioinformatic tools, has enabled exponential progress in the understanding of multiple diseases, as well as in the discovery of new biomarkers and therapeutic targets. miRNAs are non-coding RNAs that are naturally encoded in the genomes of various species and are crucially involved in regulating gene expression. In this way, the study of miRNAs has gained considerable attention in recent years as a promising approach to identifying novel biomarkers for various diseases. Cancer has been the most extensively studied field regarding miRNAs because of their significant influence on cell proliferation and gene expression, both of which are critical processes in tumorigenesis [14,15]. Transcriptomic studies have detected miRNAs in cell-free condition in blood, saliva, tears and urine, among others [16]. The dysregulation of identified miRNAs and their presence in these fluids allow miRNAs to be considered non-invasive liquid biopsies [16].

The utility of miRNAs as prognosis biomarkers, therapeutic targets and even components implicated in treatment resistance in cancer [15,17] has allowed for the development of the study of the role of miRNA in other disorders, including kidney diseases [18]. This review will explore the emerging role of miRNAs in the context of ORG, focusing on their potential as biomarkers and therapeutic targets for this increasingly prevalent disease.

## 2. MicroRNAs: Biological Functions and Roles in Obesity-Associated Pathophysiology

**Figure 1:**
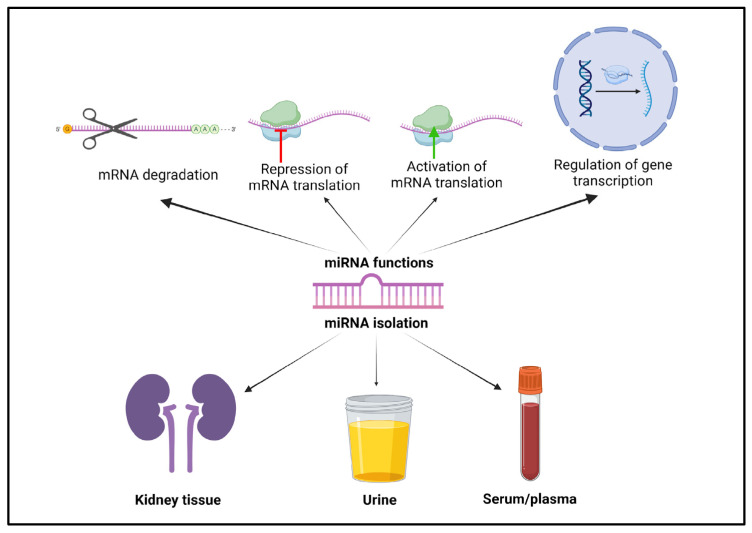
miRNA functions and isolation sources. Created in BioRender, https://BioRender.com/m51g741, accessed on 20 February 2025.

MicroRNAs (miRNAs) are a class of smallRNAs (short, non-coding RNAs) that function as critical regulators of gene expression and genome defense (Figure 1). The biogenesis of miRNAs primarily follows two pathways: canonical and non-canonical. In the canonical pathway, miRNAs are transcribed as pri-miRNAs, which are processed in the nucleus by Drosha and its cofactor DGCR8 to form pre-miRNAs [19,20,21]. These are then exported to the cytoplasm, where Dicer further processes them into a duplex RNA. The duplex is loaded onto the RISC complex, which guides the mature miRNA to its target mRNA to regulate translation [16,20]. In contrast, non-canonical pathways involve alternative processing mechanisms, independent of Drosha or Dicer, producing less common miRNA variants [19]. While only a small fraction of miRNAs are generated through these non-canonical routes, they can still play important roles in various cellular processes.

The miRNAs bind to the complementary sequence of a target mRNA, leading to its degradation. They can also block the translation machinery, thereby preventing protein synthesis, or, alternatively, they can promote protein production by activating mRNA translation. Finally, miRNAs influence the transcriptional activity of specific genes, so they are importantly involved in the regulation of gene transcription, impacting overall gene expression [22]. These diverse functions help to maintain cellular homeostasis; however, if they are pathologically up- or downregulated, they can contribute to the development of various diseases. As miRNAs are conserved between species, their dysregulation in animal models is often confirmed in humans [20]. All these characteristics of miRNAs make them potential therapeutic targets and diagnostic and prognostic biomarkers for numerous diseases, including obesity (Figure 1).

Obesity is associated with several key pathological changes, each contributing to its development and comorbidities. Insulin resistance, a sedentary lifestyle and the onset of metabolic syndrome are also closely linked to the progression of obesity [3,23,24]. An imbalance between the adipocytokines adiponectin and leptin explains the activation of the sympathetic nervous system in individuals with obesity. Regarding cellular implications, one of the most prominent shifts is the transition from M2-macrophages, which have anti-inflammatory properties, to M1-macrophages, which are pro-inflammatory [4]. This shift promotes oxidative stress and fibrosis and favors lipid accumulation, all of which exacerbate the obesity condition. Importantly, there is increasing evidence that all of these processes are regulated by miRNAs.

Pro-inflammatory pathways are predominantly involved in obesity and obesity-related comorbidities [25]. miR-519d overexpression in the subcutaneous adipose tissue of adults with obesity causes the suppression of PPARA protein, which is involved in fatty acid homeostasis and consequently causes lipid accumulation in adipocytes, triggering inflammation and favoring metabolic dysfunction [26]. miR-27a has also been reported to be overexpressed in obese mice, and if injected into lean mice, it causes glucose intolerance [27,28] and also inhibits PPARA [27]. miR-10a-5p levels are reduced in macrophages of adipose tissue after HFD, and its administration in mice inhibits inflammation and glucose intolerance [29,30]. miR-34a seems to target M2-type macrophages, stimulating chronic inflammation [31], while miR-99a is overexpressed in M2-macrophages and targets TNFα [32], which explains why miR-99a expression is negatively correlated with inflammation in human adipose tissue and obesity [33].

As obesity, insulin resistance, metabolic syndrome and diabetes sometimes form a single continuum, particular insulin resistance and obesity profiles have been described as involving specific miRNAs [34,35]. miRNA imbalance is caused by the obesity milieu and metabolic syndrome, but miRNA regulation can also predispose individuals to these pathological conditions [36]. Indeed, the injection of circulating miRNAs from obese mice into lean mice caused insulin resistance and modulated glucose and lipid metabolism [37,38]. miR-103 and miR-107 inhibit caveolin synthesis, which, in turn, promotes dysregulation of insulin receptors [39]. miR-802 is one of the miRNAs whose mechanistic effect on insulin resistance has been most thoroughly understood. It is overexpressed in the liver of both mice and humans, causing insulin resistance and glucose intolerance. It has been shown that its target is the transcription of HFN1B, which ultimately impairs insulin signaling [40]. miR-33b has been found to be significantly higher in hypercholesterolemic and obese patients, but not in those with hypercholesterolemia alone, with strong positive correlations with an increase in body mass index, low-density lipoprotein cholesterol, total cholesterol and triglycerides [41].

Although scientific research has advanced, there remains some reluctance in society to fully recognize obesity as a pathological condition. However, evidence linking differentially expressed miRNAs in obesity to other comorbidities, such as ORG [19], or even cancer risk [42], along with findings such as that exercise can improve certain altered miRNAs in this condition [43,44], supports the idea that obesity is indeed a disease with serious health consequences. Delving into the pathophysiology of obesity, as well as the dysregulated pathways involved and their triggers, is crucial to gain an understanding of the potential consequences of this condition and achieve advancements toward possible therapeutic targets. This deeper knowledge can help to raise awareness and guide progress in treatment strategies [21]. This growing body of knowledge regarding the role of miRNAs in obesity-related processes reinforces the hypothesis that they may also be involved in complications such as obesity-related glomerulopathy (ORG). Therefore, it is crucial to explore how these molecular regulators operate in the context of kidney diseases specifically.

## 3. An Overview of miRNA Changes in Different Diseases, with an Emphasis on Renal Pathologies

The rise of bioinformatic tools and next-generation sequencing has greatly expanded our ability to explore miRNA involvement across different pathologies. While much of the initial research in this field focused on cancer, recent efforts have extended into metabolic and renal diseases, including obesity-related glomerulopathy (ORG). Beyond their role in metabolic and inflammatory conditions, microRNAs (miRNAs) have also emerged as key molecular players in renal pathophysiology. Recent findings highlight that miRNA dysregulation contributes to glomerular and tubular injury, renal fibrosis and inflammatory responses—central features in chronic kidney disease (CKD), including ORG. A deeper understanding of these mechanisms may enable earlier detection and targeted therapies in kidney disorders.

Dysregulation of miRNAs has been implicated in a broad range of human diseases, including cancer, cardiovascular disorders, metabolic syndromes and inflammatory conditions. In cancer research, miRNAs have been extensively studied as biomarkers for prognosis and treatment response, given their ability to modulate tumorigenesis through the regulation of oncogenes and tumor suppressor genes. More recently, increasing attention has been directed toward the role of miRNAs in kidney diseases. Studies have demonstrated that specific miRNAs are involved in the progression of glomerular and tubular injury, renal fibrosis and inflammatory responses in nephropathies such as diabetic nephropathy, lupus nephritis, IgA nephropathy and focal segmental glomerulosclerosis (FSGS). These findings support the notion that miRNAs may serve not only as mechanistic mediators, but also as potential biomarkers and therapeutic targets in renal pathology. This broader understanding provides a strong rationale for exploring the contribution of miRNAs in obesity-related glomerulopathy (ORG), a condition where early detection and targeted intervention remain critical unmet needs.

In recent years, researchers have begun to unravel the complex interplay between miRNAs and CKD, identifying specific miRNAs associated with various types of kidney diseases. miRNAs do not encode proteins, but they can regulate gene expression. These findings indicate that the dysregulation of miRNA activity, whether through loss or gain, plays a role in the development of kidney diseases by either enhancing or inhibiting the expression of specific target genes. According to these properties, diabetic nephropathy is one of the renal pathologies in relation to which the role of miRNAs in pathophysiology has been most extensively studied [45].

The miRNA expression profile is time-dependent and tissue-specific during embryogenesis and throughout life [20]. miRNAs are not only expressed in tissue, but are also found to be differentially expressed in blood and urine (Figure 1). This fact makes miRNAs plausible non-invasive kidney disease biomarkers. As has already been proven in some cancers [14,15,46], a correlation could be established between kidney disease prognosis and the presence of specifically deregulated miRNAs. Indeed, urinary microRNAs have been evaluated as potential biomarkers of glomerulopathies. A recent study found five miRNAs with 100% specificity and sensitivity for the diagnosis of IgA nephropathy in humans [47]. Another study explored how the expression levels of miRNAs can be used to differentiate between diabetic kidney disease and focal segmental glomerulosclerosis, revealing distinct expression patterns [48]. However, the role of the identified miRNAs in these conditions’ corresponding pathology has not been clarified, nor has the presence of these miRNAs in kidney tissue been confirmed.

To investigate miRNAs as plausible therapeutic targets, there are currently some ongoing clinical trials, such as on Alport Syndrome [49] and autosomal dominant polycystic kidney disease [50]. Even so, no miRNA implicated in kidney disease has been evaluated in a phase III clinical trial yet, due to security concerns. A single miRNA has hundreds of potential mRNA targets, and multiple miRNAs can target the same mRNA [20,51]. Consequently, while potentially beneficial, targeting by miRNAs can also lead to off-target effects in non-target tissues [16]. Promisingly, other types of non-coding RNAs, small interfering RNA and siRNA-based drugs, have recently been approved by the Food and Drug Administration for the treatment of transthyretin-mediated amyloidosis and acute hepatic porphyria, respectively [18].

## 4. The Emerging Role of miRNAs in Obesity-Related Glomerulopathy

Building on the evidence that miRNAs are central regulators in both obesity and kidney disease, we now explore their specific involvement in ORG. Renal proximal tubular epithelial cells, podocytes and mesangial cells appear to be the most sensitive to lipid accumulation, due to a lack of the molecular machinery required to manage large lipid overload [19]. As a result, lipid accumulation in these cells will lead to cellular dysfunction and injury, triggering inflammation, oxidative stress, disruption of renin–angiotensin–aldosterone activity and insulin resistance. Although many miRNAs have been reported to play a role in kidney disease in murine models of diabetes and obesity, only a few have been specifically identified as involved in obesity-related kidney disease in non-diabetic murine models (Table 1).

**Figure 2:**
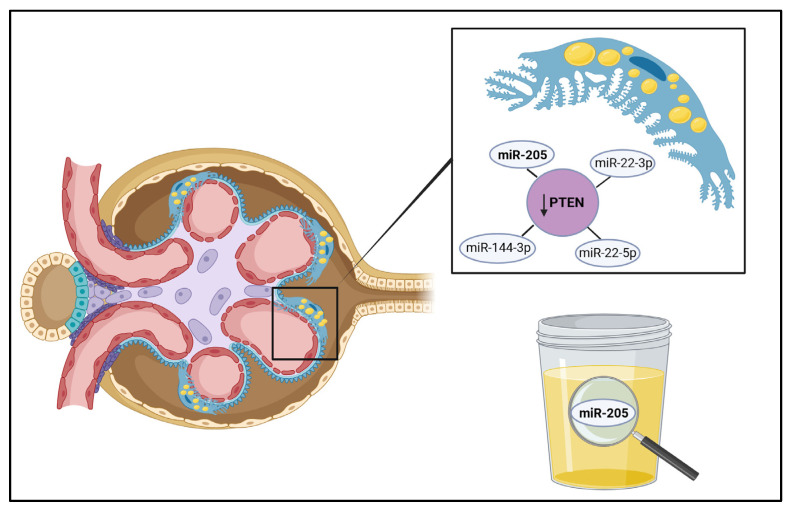
In this figure, a glomerulus in the early stages of ORG is shown. There is an increase in lipid vacuoles within the cytoplasm of the podocytes, represented as yellow circles. In this experimental model of ORG, miR-205 was differentially expressed in podocytes, where it interacts with other microRNAs depicted in the figure to promote the downregulation of PTEN. This reduction in PTEN levels facilitates the increased endocytosis of lipid vacuoles—a hallmark of the early stages of ORG. Moreover, the overexpression of miR-205 in glomeruli was also detectable in urine, highlighting its potential as a non-invasive biomarker for the disease. Created in BioRender, https://BioRender.com/s48o649, accessed on 20 February 2025.

- miR-802 [52]: The IkB kinase/NF-kB (IKK/NF-kB) signaling pathway is involved in the synthesis and secretion of chemokines. The miR-802 has previously been related with pro-inflammatory characteristics. As inflammation is one of the main cornerstones of obesity-related kidney disease development, Sun D et al. aimed to evaluate the implications of miR-802 in ORG. After 16 weeks of feeding C57BL/6J mice with normal chow (NC) or a high-fat diet (HFD), the HFD group showed significantly increased miR-802 levels in the kidneys. The increase in miR-802 was also positively related with serum levels of BUN and creatinine. Moreover, the authors demonstrated that miR-802 activated IKK/NF-kB pathways through interaction with the 3′ UTR of NF-Kn-repressing factor. Another group of obese rodents was treated with 1 × 10^9^ lentivirus particles encoding either a miR-802 sponge, which would bind and neutralize miR-802, or a control, to investigate the effects of miR-802 inhibition. Treatment with the miR-802 sponge led to a significant reduction in weight, blood urea nitrogen (BUN), inflammatory markers, fibrosis factors, glomerular size and basement membrane thickness. Finally, the circulating levels of miR-802 were higher in subjects with obesity compared with lean individuals. Although miR-802 was positively correlated with creatinine levels in humans, it was negatively correlated with creatinine clearance.
- miR-130b [53]: Adiponectin is an adipokine with anti-inflammatory properties that is usually decreased in patients with obesity. These low levels of adiponectin are thought to encourage overactivation of the renal sympathetic nervous system and also promote albuminuria through podocyte injury [4]. miR-130b has previously been related to renal fibrosis and other kidney diseases. After 16 weeks of a control diet or an HFD in adipoKO and wildtype (WT) mice with a C57BL/6J background, Pereira BMV et al. confirmed that there was overexpression of miR-130b in the kidney of the group of adipoKO mice with an HFD. Both adipoKO and WT mice fed with an HFD showed non-detectable levels of adiponectin compared with groups. However, an in vitro analysis of cultured podocytes of WT mice disproved the adiponectin treatment’s regulation of miR-130 expression [53].
- miR-21 [54]: In order to study the role of inflammation, fibrosis and adiponectin in obesity-related kidney disease, Morrison MC et al. fed a human CRP transgenic (huCRPtg) mouse model with a C57BL/6J background with an NC diet or an HFD for 50 weeks, and also evaluated the effects of rosiglitazone and rosuvastatin based on their anti-inflammatory effects. Since miR-21 has previously been associated with fibrosis, inflammation and albuminuria, its expression was also measured. Renal miR-21 was overexpressed in the HFD group compared with the NC group. Also, miR-21 expression was significantly correlated with the kidney damage marker KIM-1 and with renal fibrosis, while no such correlations were found for adiponectin levels or exposure.
- miR-155 [55]: Circulating miR-155 has already been associated with eGFR and proteinuria in CKD, and with histological kidney damage in patients with IgA nephropathy. Zheng C et al. fed C57BL/6J mice with either an HFD or a control diet (CD) for 20 weeks. Afterwards, the HFD mice were injected with 1 × 10^10^ IU viral particles of lentivirus encoding the miR-155 sponge or a control vector. To silence SHIP1/INNP5D, which is a negative regulator of inflammatory response in the NF-κB signaling pathway, 1 × 10^10^ IU viral particles of lentivirus encoding INPP5D siRNA were also injected. Four weeks after these infusions, renal miR-155 was upregulated 4-fold in HFD mice in comparison to CD mice. There was a linear correlation between renal miR-155 and albuminuria and BUN. Those HFD mice treated with the miR-155 sponge showed attenuation of glomerular enlargement, fibrosis and tubular damage, blocked macrophage infiltration and lipid deposits, and decreased albuminuria and BUN, compared with the control vector-treated HFD mice. The authors were able to confirm that miR-155 significantly decreased the luciferase activity in INPP5D 3′-UTR transfected cells, and real-time PCR analysis showed that the gene level of INPP5D was significantly decreased by overexpression of miR-155 in the kidneys. Finally, the suppression of INPP5D increased the levels of albuminuria and BUN and significantly increased glomerular size; all of this was accompanied by renal inflammatory response and oxidative stress. In summary, miR-155 seems to contribute to ORG development by promoting inflammation and oxidative stress through inhibition of the INNP5D signaling pathway.
- miR-205 [8]: The PTEN gene (phosphatase and tensin homolog) is essential in kidney health, particularly in relation to inflammation and podocyte injury, in which case it is regulated by various miRNAs [56,57]. Its inhibition has been associated with several obesity-related pathways, including enhanced muscle regeneration [58], mitochondrial dysfunction [59], endoplasmic reticulum stress [60], increased renal inflammation and fibrosis [61] and the promotion of insulin resistance [62]. Interestingly, podocytes in patients with ORG have shown increased lipid endocytosis, which has been linked to reduced PTEN expression [63]. In this model, Wistar Han rats were fed with a standard diet or an HFD for 10 weeks. It was proven that the downregulation of PTEN in the kidneys of the HFD group was driven by interactions between miR-205, miR-22-3p, miR-22-5p and miR-144-3p, and was associated with increased lipid uptake in podocytes (Figure 2). Notably, miR-205 was not only upregulated in the kidneys of rats with ORG, but was also found to be differentially expressed in their urine (Figure 2). Furthermore, the increase in the mesangial matrix and podocyte hypertrophy observed in these animals correlated with changes in miRNA expression in both kidney tissue and urine.

## 5. Future Perspectives and Conclusions

Obesity is one of the main threats to the health of today’s society. It is a growing cause of kidney disease, highlighting the need for better biomarkers for early detection and treatment. Analyzing and studying the role of miRNAs in the development of obesity and its comorbidities can help to uncover methods of prevention. A deeper understanding of these molecular mechanisms holds great promise for advancing kidney health. The isolation of miRNAs in blood or urine that have been shown to be involved in the development of specific diseases would open up promising new directions for research on their use as biomarkers. Recent advances in miRNA research have revealed their potential as key regulators in the development and progression of obesity and kidney diseases, which facilitates the study of potential treatments and disease prognosis. Delving into the role of miRNAs in the development of a disease helps to improve our understanding of the disease’s pathophysiology and the involved pathways.

Despite these findings, only a limited number of miRNAs have been identified with their target genes in ORG (Table 1). Some miRNAs, such as miR-802, miR-130b, miR-21 and miR-155, isolated in kidney tissue, have been shown to promote inflammation and fibrosis in kidney cells in experimental ORG models. These miRNAs contribute to the progression of ORG by regulating pathways involved in kidney damage, such as the NF- κB signaling pathway. Notably, miR-205 regulates PTEN expression in the kidney, contributing to increased lipid uptake in podocytes during the early stages of ORG, and miR-205 can also be isolated in urine in these cases.

Moving forward, developing miRNA-based therapies will require the identification of specific miRNA targets and further exploration of their roles through patient sample analysis. However, challenges remain in miRNAs’ interactions and their potential off-target effects, which must be addressed in future clinical trials. All in all, miRNAs represent a promising frontier for advancing the diagnosis and treatment of both general kidney diseases and ORG specifically.

## Figures and Tables

**Table 1:** Summary of miRNAs involved in obesity-related glomerulopathy.

	Target Gene	Regulation Status	Involvement with ORG Development	Model Used	Source of Isolation	REFERENCE
**miR-802**	Inhibition of NF-κB-repressing factor (NRF)	Upregulated	Promotes kidney inflammation and fibrosis	C57BL/6J mice and humans	Kidney tissue (mice); serum (humans)	Sun et al. [52] (PMID 30729676)
**miR-130b**	Not identified, correlated with decreased adiponectin	Upregulated	Promotes renal lipid accumulation and albuminuria	adipoKO mice on a C57BL/6 background	Kidney tissue	Pereira et al. [53] (PMID 32652137)
**miR-21**	Not identified, highly correlated with expression of KIM-1	Upregulated	Promotes kidney inflammation and fibrosis	HuCRPtg mice on a C57BL/6 background	Kidney tissue	Morrison et al. [54] (PMID 28588299)
**miR-155**	Inhibition of SHIP1/INNP5D (NF-κB signaling pathway)	Upregulated	Promotes kidney inflammation and oxidative stress	C57BL/6J mice and humans	Kidney tissue	Zheng et al. [55] (PMID 30715692)
**miR-205**	Inhibition of PTEN	Upregulated	Promotes increased lipid uptake in podocytes	Wistar Han rats	Kidney tissue and urine	López-Martínez et al. [8] (PMID 38928144)

## Data Availability

No new data were generated or analyzed during this study.

## Abbreviations

The following abbreviations are used in this manuscript:

ORG	Obesity-related glomerulopathy
CKD	Chronic kidney disease
miRNAs	MicroRNAs
mRNA	Messenger RNA
BUN	Blood urea nitrogen
CD	Control diet
PTEN	Phosphatase and tensin homolog
SHIP1	SH2-containing inositol phosphatase 1
INNP5D	Inositol Polyphosphate-5-Phosphatase D
siRNA	Small interfering RNA

## References

[1] Weisinger J.R., Kempson R.L., Eldridge F.L., Swenson R.S. (1974). The Nephrotic Syndrome: A Complication of Massive Obesity. Ann. Intern. Med..

[2] Cohen A.H. (1975). Massive obesity and the kidney. A morphologic and statistical study. Am. J. Pathol..

[3] Kambham N., Markowitz G.S., Valeri A.M., Lin J., D’agati V.D. (2001). Obesity-related glomerulopathy: An emerging epidemic. Kidney Int..

[4] D’Agati V.D., Chagnac A., De Vries A.P.J., Levi M., Porrini E., Herman-Edelstein M., Praga M. (2016). Obesity-related glomerulopathy: Clinical and pathologic characteristics and pathogenesis. Nat. Rev. Nephrol..

[5] Bikbov B., Purcell C.A., Levey A.S., Smith M., Abdoli A., Abebe M., Adebayo O.M., Afarideh M., Agarwal S.K., Agudelo-Botero M. (2020). Global, regional, and national burden of chronic kidney disease, 1990–2017: A systematic analysis for the Global Burden of Disease Study 2017. Lancet.

[6] Sowers J.R. (2003). Obesity as a cardiovascular risk factor. Am. J. Med..

[7] Serra A., Romero R., Lopez D., Navarro M., Esteve A., Perez N., Alastrue A., Ariza A. (2008). Renal injury in the extremely obese patients with normal renal function. Kidney Int..

[8] López-Martínez M., Armengol M.P., Pey I., Farré X., Rodríguez-Martínez P., Ferrer M., Porrini E., Luis-Lima S., Díaz-Martín L., Rodríguez-Rodríguez A.E. (2024). Integrated miRNA–mRNA Analysis Reveals Critical miRNAs and Targets in Diet-Induced Obesity-Related Glomerulopathy. Int. J. Mol. Sci..

[9] Jung C.H., Lee M.J., Kang Y.M., Hwang J.Y., Kim E.H., Park J.-Y., Kim H.-K., Lee W.J. (2015). The risk of chronic kidney disease in a metabolically healthy obese population. Kidney Int..

[10] Bancu I., Navarro Díaz M., Serra A., Granada M., Lopez D., Romero R., Bonet J. (2016). Low Insulin-Like Growth Factor-1 Level in Obesity Nephropathy: A New Risk Factor?. PLoS ONE.

[11] López-Martínez M., Luis-Lima S., Morales E., Navarro-Díaz M., Negrín-Mena N., Folgueras T., Escamilla B., Estupiñán S., Delgado-Mallén P., Marrero-Miranda D. (2020). The estimation of GFR and the adjustment for BSA in overweight and obesity: A dreadful combination of two errors. Int. J. Obes..

[12] Azushima K., Gurley S.B., Coffman T.M. (2018). Modelling diabetic nephropathy in mice. Nat. Rev. Nephrol..

[13] Teixido-Trujillo S., Luis-Lima S., López-Martínez M., Navarro-Díaz M., Díaz-Martín L., Escasany-Martínez E., Gaspari F., Rodríguez-Rodríguez A.E. (2023). Measured GFR in murine animal models: Review on methods, techniques, and procedures. Pflugers Arch..

[14] Rupaimoole R., Slack F.J. (2017). MicroRNA therapeutics: Towards a new era for the management of cancer and other diseases. Nat. Rev. Drug Discov..

[15] Cavaliere A.F., Perelli F., Zaami S., Piergentili R., Mattei A., Vizzielli G., Scambia G., Gianluca S., Restaino S., Signore F. (2021). Towards Personalized Medicine: Non-Coding RNAs and Endometrial Cancer. Healthcare.

[16] Seyhan A.A. (2023). Circulating microRNAs as Potential Biomarkers in Pancreatic Cancer—Advances and Challenges. Int. J. Mol. Sci..

[17] Wang C., Wu C., Yang Q., Ding M., Zhong J., Zhang C.-Y., Ge J., Wang J., Zhang C. (2016). miR-28-5p acts as a tumor suppressor in renal cell carcinoma for multiple antitumor effects by targeting RAP1B. Oncotarget.

[18] Seyhan A.A. (2024). Trials and Tribulations of MicroRNA Therapeutics. Int. J. Mol. Sci..

[19] Caus M., Eritja À., Bozic M. (2021). Role of micrornas in obesity-related kidney disease. Int. J. Mol. Sci..

[20] Mahtal N., Lenoir O., Tinel C., Anglicheau D., Tharaux P.-L. (2022). MicroRNAs in kidney injury and disease. Nat. Rev. Nephrol..

[21] Ji C., Guo X. (2019). The clinical potential of circulating microRNAs in obesity. Nat. Rev. Endocrinol..

[22] Carthew R.W., Sontheimer E.J. (2009). Origins and Mechanisms of miRNAs and siRNAs. Cell.

[23] Martínez-Montoro J.I., Morales E., Cornejo-Pareja I., Tinahones F.J., Fernández-García J.C. (2022). Obesity-related glomerulopathy: Current approaches and future perspectives. Obes. Rev..

[24] Chen Y., Dabbas W., Gangemi A., Benedetti E., Lash J., Finn P.W., Perkins D.L. (2021). Obesity Management and Chronic Kidney Disease. Semin Nephrol..

[25] Kiran S., Kumar V., Kumar S., Price R.L., Singh U.P. (2021). Adipocyte, Immune Cells, and miRNA Crosstalk: A Novel Regulator of Metabolic Dysfunction and Obesity. Cells.

[26] Martinelli R., Nardelli C., Pilone V., Buonomo T., Liguori R., Castanò I., Buono P., Masone S., Persico G., Forestieri P. (2010). miR-519d overexpression is associated with human obesity. Obesity.

[27] Chen T., Zhang Y., Liu Y., Zhu D., Yu J., Li G., Sun Z., Wang W., Jiang H., Hong Z. (2019). MiR-27a promotes insulin resistance and mediates glucose metabolism by targeting PPAR-γ-mediated PI3K/AKT signaling. Aging.

[28] Zhang Y., Qian B., Yang Y., Niu F., Lin C., Yuan H., Wang J., Wu T., Shao Y., Shao S. (2024). Visceral Adipocyte-Derived Extracellular Vesicle miR-27a-5p Elicits Glucose Intolerance by Inhibiting Pancreatic β-Cell Insulin Secretion. Diabetes.

[29] Cho Y.K., Son Y., Kim S.-N., Song H.-D., Kim M., Park J.-H., Jung Y.-S., Ahn S.-Y., Saha A., Granneman J.G. (2019). MicroRNA-10a-5p regulates macrophage polarization and promotes therapeutic adipose tissue remodeling. Mol. Metab..

[30] Lee S., Cho Y.K., Kim H., Choi C., Kim S., Lee Y.-H. (2024). miR-10a regulates cell death and inflammation in adipose tissue of male mice with diet-induced obesity. Mol. Metab..

[31] Zhou J., Li Z., Wu T., Zhao Q., Zhao Q., Cao Y. (2020). LncGBP9/miR-34a axis drives macrophages toward a phenotype conducive for spinal cord injury repair via STAT1/STAT6 and SOCS3. J. Neuroinflammation.

[32] Jaiswal A., Reddy S.S., Maurya M., Maurya P., Barthwal M.K. (2019). MicroRNA-99a mimics inhibit M1 macrophage phenotype and adipose tissue inflammation by targeting TNFα. Cell Mol Immunol..

[33] Maurya M., Barthwal M.K. (2021). MicroRNA-99a: A potential double-edged sword targeting macrophage inflammation and metabolism. Cell Mol. Immunol..

[34] Jones A., Danielson K.M., Benton M.C., Ziegler O., Shah R., Stubbs R.S., Das S., Macartney-Coxson D. (2017). miRNA Signatures of Insulin Resistance in Obesity. Obesity.

[35] Agbu P., Carthew R.W. (2021). MicroRNA-mediated regulation of glucose and lipid metabolism. Nat. Rev. Mol. Cell Biol..

[36] Zhang Y., Chen A., Lu S., Liu D., Xuan X., Lei X., Zhong M., Gao F. (2025). Noncoding RNA profiling in omentum adipose tissue from obese patients and the identification of novel metabolic biomarkers. Front. Genet..

[37] Ying W., Riopel M., Bandyopadhyay G., Dong Y., Birmingham A., Seo J.B., Ofrecio J.M., Wollam J., Hernandez-Carretero A., Fu W. (2017). Adipose Tissue Macrophage-Derived Exosomal miRNAs Can Modulate In Vivo and In Vitro Insulin Sensitivity. Cell.

[38] Castaño C., Kalko S., Novials A., Párrizas M. (2018). Obesity-associated exosomal miRNAs modulate glucose and lipid metabolism in mice. Proc. Natl. Acad. Sci. USA.

[39] Trajkovski M., Hausser J., Soutschek J., Bhat B., Akin A., Zavolan M., Heim M.H., Stoffel M. (2011). MicroRNAs 103 and 107 regulate insulin sensitivity. Nature.

[40] Kornfeld J.-W., Baitzel C., Könner A.C., Nicholls H.T., Vogt M.C., Herrmanns K., Scheja L., Haumaitre C., Wolf A.M., Knippschild U. (2013). Obesity-induced overexpression of miR-802 impairs glucose metabolism through silencing of Hnf1b. Nature.

[41] Masoumi-Ardakani Y., Eghbalian M., Fallah H., Jafari A., Shahouzehi B. (2025). Exploring serum miR-33b as a novel diagnostic marker for hypercholesterolemia and obesity: Insights from a pilot case-control study. BMC Endocr. Disord..

[42] Hanusek K., Karczmarski J., Litwiniuk A., Urbańska K., Ambrozkiewicz F., Kwiatkowski A., Martyńska L., Domańska A., Bik W., Paziewska A. (2022). Obesity as a Risk Factor for Breast Cancer—The Role of miRNA. Int. J. Mol. Sci..

[43] Silveira A., Gomes J., Roque F., Fernandes T., de Oliveira E.M. (2022). MicroRNAs in Obesity-Associated Disorders: The Role of Exercise Training. Obes. Facts..

[44] Fernandes T., Casaes L., Soci Ú., Silveira A., Gomes J., Barretti D., Roque F., Oliveira E. (2018). Exercise Training Restores the Cardiac Microrna-16 Levels Preventing Microvascular Rarefaction in Obese Zucker Rats. Obes. Facts.

[45] Szostak J., Gorący A., Durys D., Dec P., Modrzejewski A., Pawlik A. (2023). The Role of MicroRNA in the Pathogenesis of Diabetic Nephropathy. Int. J. Mol. Sci..

[46] Andersen C.L., Jensen J.L., Falck Ørntoft T. (2004). Normalization of Real-Time Quantitative Reverse Transcription-PCR Data: A Model-Based Variance Estimation Approach to Identify Genes Suited for Normalization, Applied to Bladder and Colon Cancer Data Sets. Cancer Res..

[47] Shankar M., Shetty A., N.S. M., C.G. S., A. K., Tennankore K. (2023). Urinary exosomal miRNA signature of IgA nephropathy: A case-control study. Sci. Rep..

[48] Trabulus S., Zor M.S., Alagoz S., Dincer M.T., Meşe M., Yilmaz E., Turanli E.T., Seyahi N. (2024). Profiling of five urinary exosomal miRNAs for the differential diagnosis of patients with diabetic kidney disease and focal segmental glomerulosclerosis. PLoS ONE.

[49] Gomez I.G., MacKenna D.A., Johnson B.G., Kaimal V., Roach A.M., Ren S., Nakagawa N., Xin C., Newitt R., Pandya S. (2015). Anti-microRNA-21 oligonucleotides prevent Alport nephropathy progression by stimulating metabolic pathways. J. Clin. Investig..

[50] Bais T., Gansevoort R.T., Meijer E. (2022). Drugs in Clinical Development to Treat Autosomal Dominant Polycystic Kidney Disease. Drugs.

[51] Ru Y., Kechris K.J., Tabakoff B., Hoffman P., Radcliffe R.A., Bowler R., Mahaffey S., Rossi S., Calin G.A., Lynne Bemis L. (2014). The multiMiR R package and database: Integration of microRNA-target interactions along with their disease and drug associations. Nucleic Acids Res..

[52] Sun D., Chen J., Wu W., Tang J., Luo L., Zhang K., Jin L., Lin S., Gao Y., Yan X. (2019). MiR-802 causes nephropathy by suppressing NF-κB-repressing factor in obese mice and human. J. Cell Mol. Med..

[53] Pereira B.M.V., Thieme K., de Araújo L., Rodrigues A.C. (2020). Lack of adiponectin in mice accelerates high-fat diet-induced progression of chronic kidney disease. Life Sci..

[54] Morrison M.C., Yakala G.K., Liang W., Wielinga P.Y., Salic K., van Koppen A., Tomar T., Kleemann R., Heeringa P., Kooistra T. (2017). Protective effect of rosiglitazone on kidney function in high-fat challenged human-CRP transgenic mice: A possible role for adiponectin and miR-21?. Sci. Rep..

[55] Zheng C., Zhang J., Chen X., Zhang J., Ding X., You X., Fan L., Chen C., Zhou Y. (2019). MicroRNA-155 Mediates Obesity-Induced Renal Inflammation and Dysfunction. Inflammation.

[56] Ren Q., Yu S., Zeng H., Xia H. (2022). The role of PTEN in puromycin aminonucleoside-induced podocyte injury. Int. J. Med. Sci..

[57] Duan P., Tan J., Miao Y., Zhang Q. (2022). PINK1/Parkin-Mediated Mitophagy Plays a Protective Role in Albumin Overload-Induced Renal Tubular Cell Injury. Front. Biosci..

[58] Hu Z., Wang H., Lee I.H., Modi S., Wang X., Du J., Mitch W.E. (2010). PTEN inhibition improves muscle regeneration in mice fed a high-fat diet. Diabetes.

[59] Ji J., Qin Y., Ren J., Lu C., Wang R., Dai X., Zhou R., Huang Z., Xu M., Chen M. (2015). Mitochondria-related miR-141-3p contributes to mitochondrial dysfunction in HFD-induced obesity by inhibiting PTEN. Sci. Rep..

[60] Garner K.L., Betin V.M.S., Pinto V., Graham M., Abgueguen E., Barnes M., Bedford D.C., McArdle C.A., Coward R.J.M. (2018). Enhanced insulin receptor, but not PI3K, signalling protects podocytes from ER stress. Sci. Rep..

[61] An C., Jiao B., Du H., Tran M., Zhou D., Wang Y. (2022). Myeloid PTEN deficiency aggravates renal inflammation and fibrosis in angiotensin II-induced hypertension. J. Cell Physiol..

[62] Axelrod C.L., Fealy C.E., Erickson M.L., Davuluri G., Fujioka H., Dantas W.S., Huangc E., Pergolaa K., Meya J.T., King W.T. (2021). Lipids activate skeletal muscle mitochondrial fission and quality control networks to induce insulin resistance in humans. Metabolism.

[63] Shi Y., Wang C., Zhou X., Li Y., Ma Y., Zhang R., Li R. (2020). Downregulation of PTEN promotes podocyte endocytosis of lipids aggravating obesity-related glomerulopathy. Am. J. Physiol. Renal Physiol..

